# Antiproliferative effects of mitochondria-targeted *N*-acetylcysteine and analogs in cancer cells

**DOI:** 10.1038/s41598-023-34266-w

**Published:** 2023-05-04

**Authors:** Gang Cheng, Micael Hardy, Balaraman Kalyanaraman

**Affiliations:** 1 Department of Biophysics, Medical College of Wisconsin, 8701 Watertown Plank Road, Milwaukee, WI 53226 USA; 2 CNRS, ICR, UMR 7273, Aix Marseille Univ, 13013 Marseille, France

**Keywords:** Cancer metabolism, Cancer metabolism

## Abstract

*N*-acetylcysteine (NAC) has been used as an antioxidant drug in tumor cells and preclinical mice tumor xenografts, and it improves adaptive immunotherapy in melanoma. NAC is not readily bioavailable and is used in high concentrations. The effects of NAC have been attributed to its antioxidant and redox signaling role in mitochondria. New thiol-containing molecules targeted to mitochondria are needed. Here, mitochondria-targeted NAC with a 10-carbon alkyl side chain attached to a triphenylphosphonium group (Mito_10_-NAC) that is functionally similar to NAC was synthesized and studied. Mito_10_-NAC has a free sulfhydryl group and is more hydrophobic than NAC. Mito_10_-NAC is nearly 2000-fold more effective than NAC in inhibiting several cancer cells, including pancreatic cancer cells. Methylation of NAC and Mito_10_-NAC also inhibited cancer cell proliferation. Mito_10_-NAC inhibits mitochondrial complex I-induced respiration and, in combination with monocarboxylate transporter 1 inhibitor, synergistically decreased pancreatic cancer cell proliferation. Results suggest that the antiproliferative effects of NAC and Mito_10_-NAC are unlikely to be related to their antioxidant mechanism (i.e., scavenging of reactive oxygen species) or to the sulfhydryl group-dependent redox modulatory effects.

## Introduction

*N*-acetylcysteine (NAC) was first approved as a drug to treat excessive mucous production in respiratory diseases (e.g., cystic fibrosis) in 1963^1,2^. Later, it was used to counteract paracetamol (i.e., acetaminophen or Tylenol) poisoning^2^. NAC also has been used as a direct scavenger of reactive oxygen species (particularly hydrogen peroxide) and as an antioxidant in cancer biology and immuno-oncology. NAC is frequently used antioxidant drugs in studies employing tumor cells, immune cells, and preclinical mouse models^3–9^. In both in vitro and in vivo studies, NAC is used in high concentrations as its bioavailability is relatively low^10,11^. Reports indicate that the effect of NAC is cancer cell dependent and stage specific^12^. NAC is membrane-permeant and crosses the blood–brain barrier depending on the dose and administration^13,14^. The effects of NAC are attributed to its thiol modulation in cells^15–17^.

**Figure 1:**
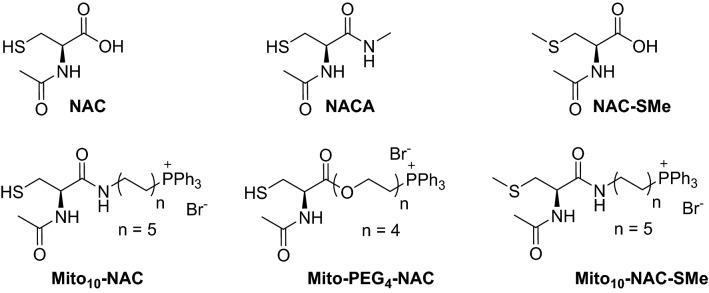
Structures of NAC and Mito_10_-NAC analogs.

The objective of this study is to determine the effect of mitochondria-targeted thiol in cancer cell proliferation. Numerous reports have shown in both in vitro and in vivo cancer studies that conjugation of drugs to a triphenylphosphonium (TPP^+^) moiety linked through an alkyl side chain selectively target mitochondria of cancer cells more so than normal cells^18,19^. The more negative mitochondrial membrane potential of cancer cells as compared with control, nontransformed cells is responsible for enhanced uptake and retention of positively charged drugs conjugated to TPP^+^^20,21^. Enhanced accumulation of TPP^+^-modified drugs (e.g*.*, Mito-vitamin-E) in tumor tissues was observed in mice xenograft administered with the drug^22^. Thus, mitochondria-targeted NAC (Mito_10_-NAC) was synthesized by attaching an alkyl side chain containing a TPP^+^ moiety (Fig. 1). Mito_10_-NAC has a free sulfhydryl group, so the molecule should exhibit similar antioxidant- and redox-modulating properties^23,24^. Age-related mitochondrial decline was attributed to a breakdown in intracellular amino acid homeostasis, cysteine in particular^24^. Cysteine is most toxic for mitochondria, and elevated non-vacuolar cysteine impairs mitochondrial respiration.

In this study, we have compared the relative antiproliferative potencies of NAC, Mito_10_-NAC, and their methylated analogs in several cancer cells. Results show that Mito_10_-NAC is nearly 1500–2000 times more potent than NAC, and that methylation of the free sulfhydryl group enhanced its antiproliferative effect (the half maximal inhibitory concentration [IC_50_] for Mito_10_-MeNAC is 1.9 µM compared with 9.6 µM for Mito_10_-NAC), indicating that the antiproliferative effect is not related to the antioxidant or radical scavenging mechanism.

## Results

### Antiproliferative effects of Mito_10_-NAC

**Figure 2:**
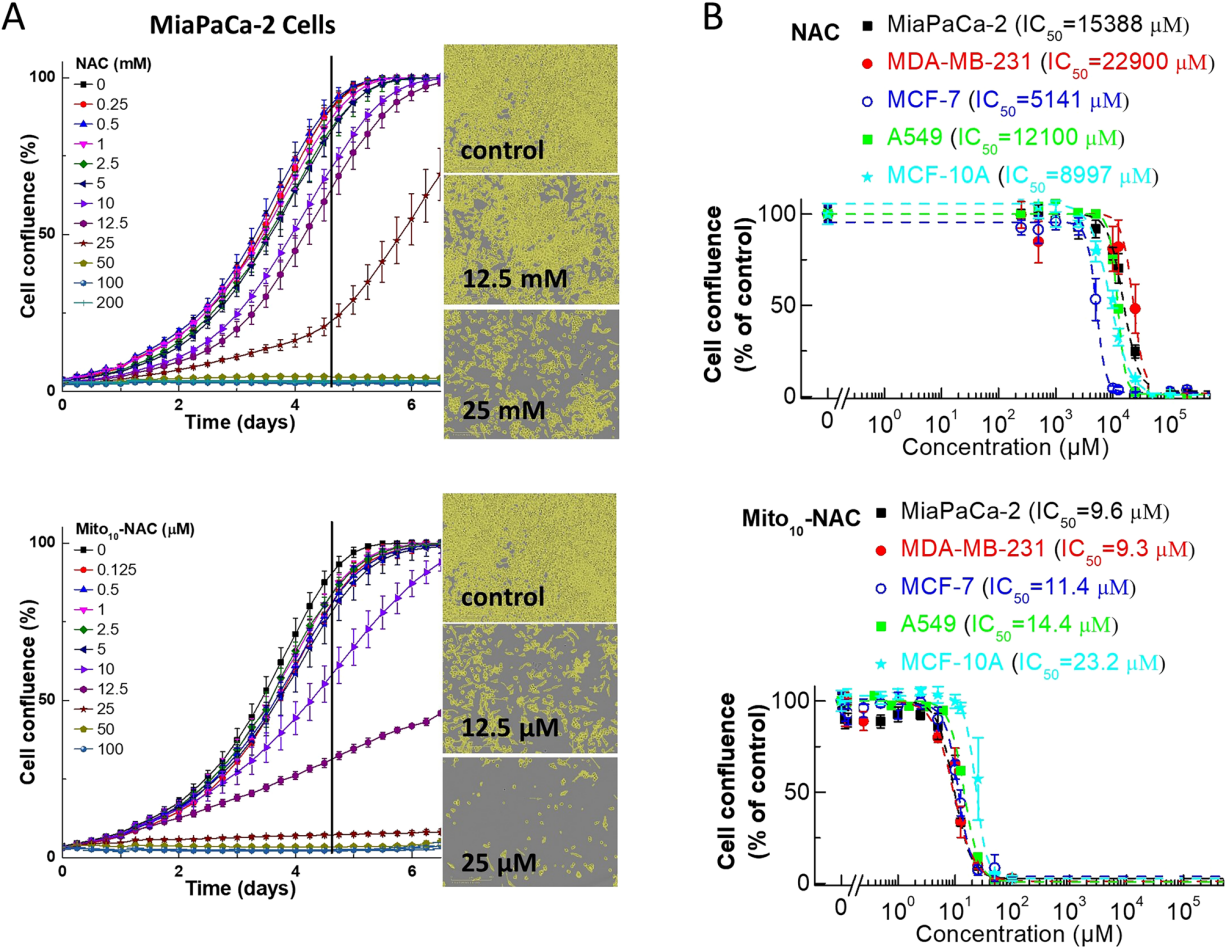
Effects of NAC and Mito_10_-NAC on the proliferation of cells derived from various cancers. (**A**) The effects of NAC and Mito_10_-NAC on the proliferation of MiaPaCa-2 cells were monitored in the IncuCyte Live-Cell Analysis System. The IncuCyte analyzer provides real-time updates on cell confluence, based on segmentation of high definition-phase contrast images. Representative cell images were shown as segmentation mask illustrated in brown when control cells reached 90% confluence (vertical solid black line). (**B**) The same proliferation monitoring methods were used for all cell lines as indicated. The IC_50_ values were determined at the point at which control cells reached ~ 90% confluence. Relative cell confluence (control is taken as 100%) is plotted against concentration. Dashed lines represent the fitting curves used to determine the IC_50_ values as indicated. Data shown are the mean ± SD, n = 4.

**Figure 3:**
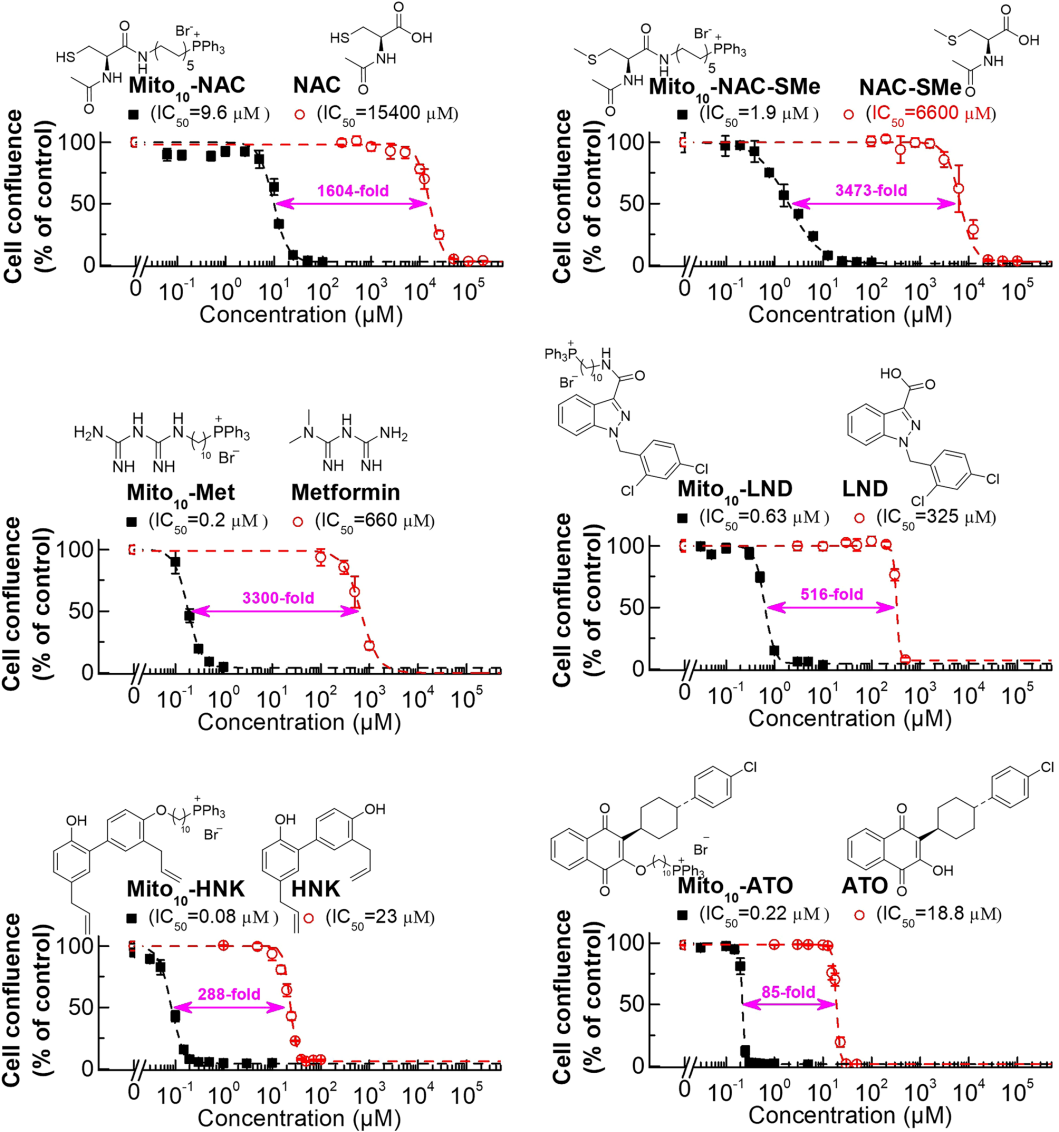
Comparisons of mitochondria-targeted drugs and the corresponding parent compounds on cell proliferation inhibitions in human pancreatic cancer (MiaPaCa-2) cells. The effects of mitochondria-targeted drugs and their parental compounds on the proliferation of MiaPaCa-2 cells were monitored in the IncuCyte Live-Cell Analysis System. The IC_50_ values were determined at the point at which control cells reached ~ 90% confluence. Relative cell confluence (control is taken as 100%) is plotted against concentration. Dashed lines represent the fitting curves used to determine the IC_50_ values as indicated. The folder of differences as indicated were calculated by the potency difference of the IC_50_ values between each mitochondria-targeted drug and its parental compound. The IC_50_ values of Mito-Met and metformin were published previously in *Cancer Research* (Cheng et al. Cancer Res, 2016). The IC_50_ values of Mito-ATO and ATO were published in *Scientific Reports* (Cheng et al. Scientific Reports, 2020; Cheng et al. Scientific Reports, 2022).

We determined the effects of Mito_10_-NAC and NAC on the proliferation of several cancer cell lines derived from pancreatic, breast, and lung cancers (MiaPaCa-2, MDA-MB-231, MCF-7, and A549) and a nonmalignant breast cancer cell line (MCF-10A) as a control. Specifically, we tested the effects of Mito_10_-NAC on the proliferation of MiaPaCa-2, MDA-MB-231, MCF-7, A549, and MCF-10A cells. Figure 2 shows the comparative effects of NAC and Mito_10_-NAC on the proliferation of these cells. Typically, mitochondria-targeted TPP^+^ drugs inhibit proliferation of cancer cells by 100–500-fold as compared with untargeted parent drugs (Fig. 3)^18^. Surprisingly, Mito_10_-NAC inhibited the proliferation of cancer cells 1500–2400 fold greater than NAC (Figs. 2 and 3). This is a totally unexpected finding. This magnitude of differential effect induced by TPP^+^-containing drugs in cancer cells is unique and, to our knowledge, has only been reported for one other TPP^+^-targeted drug^18^. One of the reasons for this considerable increase in the antiproliferative effects of Mito_10_-NAC may be related to the relative hydrophobicity difference between the untargeted and TPP^+^-conjugated analogs. Hydrophobicity calculations show that NAC is exceedingly hydrophilic, and Mito_10_-NAC is relatively more hydrophobic (log *p* values for NAC and Mito_10_-NAC are − 0.7 and 6.4, respectively) (Table 1).

**Table 1:**
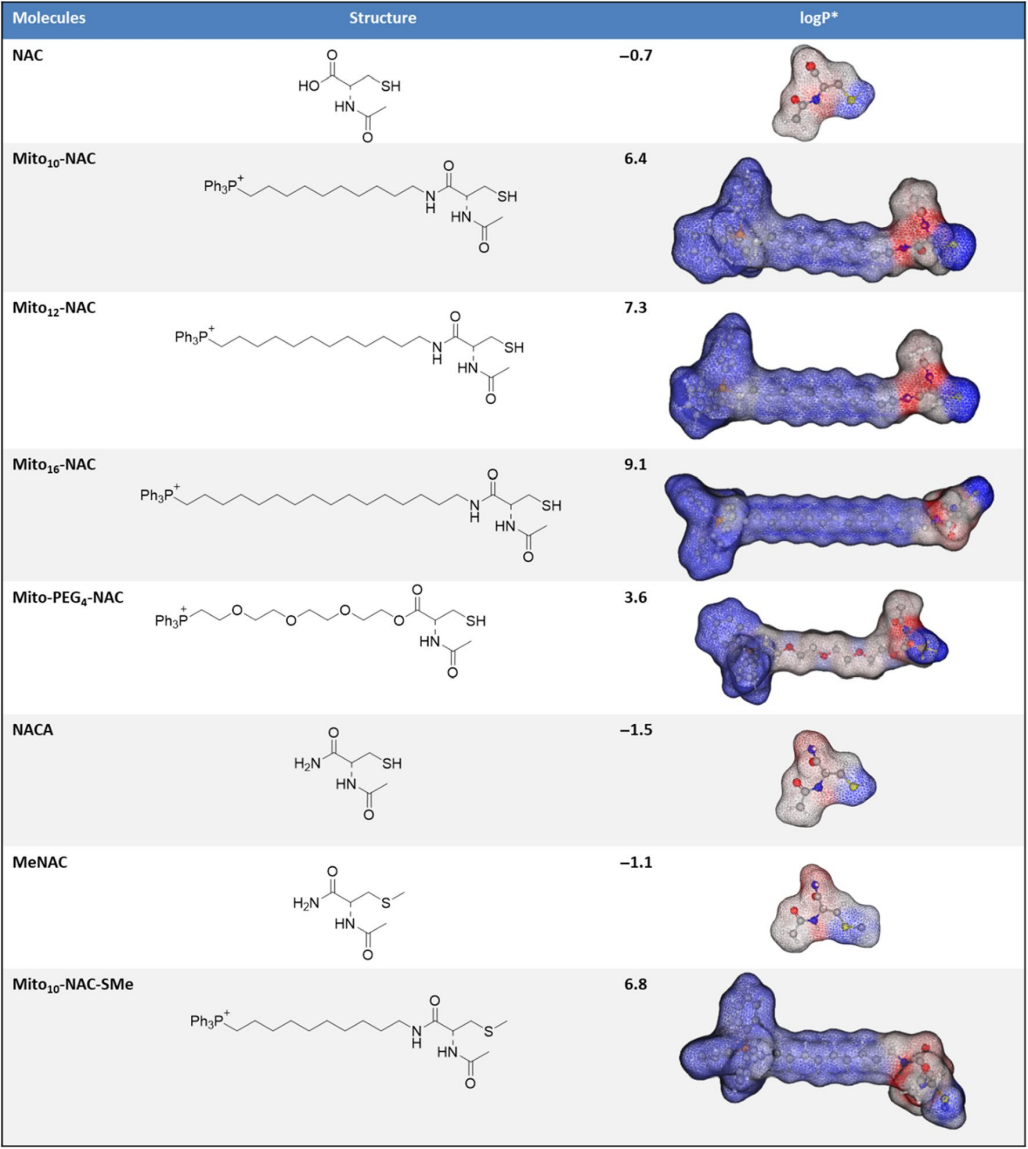
Calculated partition coefficients and relative hydrophobic regions in NAC and Mito_10_-NAC analogs.

To ensure that cell proliferation results were not affected by Mito-NAC degradation over the time course, we compared results from cell proliferation experiments where Mito-NAC was added as a bolus or added freshly every 48 h. No significant differences in cell proliferation profiles were observed, indicating that Mito-NAC remained relatively stable during the course of the experiment (Fig. S3).

Results also suggest that the NAC-induced antiproliferative effects observed at very high concentrations in cells may involve “off-target” effects and not mitochondria. At present, we do not understand the mechanism of the “off-target” effects. Results obtained from oxygen consumption experiments in MiaPaCa-2 cells also support this conclusion (see below, Fig. 6). Also, we should caution that the MCF-10A cells that were used as a control nonmalignant cell line for breast cancer are not an effective or proper nonmalignant cell line control for other types of cancer.

**Figure 4:**
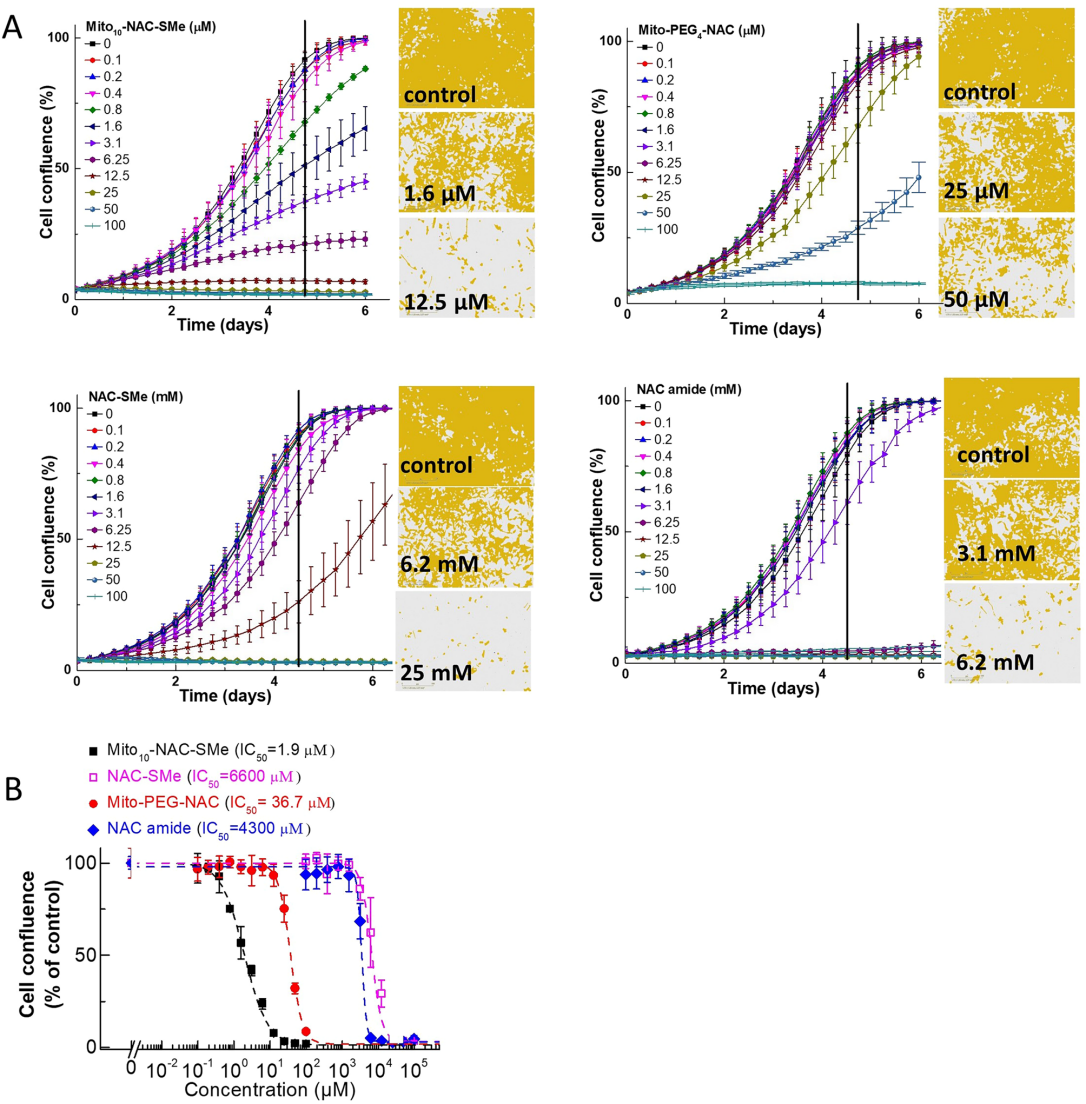
Effects of Mito_10_-NAC analogs on the proliferation of human pancreatic cancer (MiaPaCa-2) cells. (**A**) The effects of Mito_10_-NAC-SMe, Mito_10_-PEG-NAC, NAC-SMe, and NAC amide on the proliferation of MiaPaCa-2 cells were monitored in the IncuCyte Live-Cell Analysis System. The IncuCyte analyzer provides real-time updates on cell confluence, based on segmentation of high definition-phase contrast images. Representative cell images were shown as segmentation mask illustrated in brown when control cells reached 90% confluence (vertical solid black line). (**B**) The IC_50_ values were determined at the point at which control cells reached ~ 90% confluence. Relative cell confluence (control is taken as 100%) is plotted against concentration. Dashed lines represent the fitting curves used to determine the IC_50_ values as indicated. Data shown are the mean ± SD, n = 4.

We determined the effects of methyl-substituted NAC and Mito_10_-NAC on the proliferation of human pancreatic cancer (MiaPaCa-2) cells (Fig. 4). Results indicate that methylation of the sulfhydryl group actually enhanced the antiproliferative effect. This suggests that the antioxidant mechanism related to scavenging of reactive oxygen species (superoxide and hydrogen peroxide) or reactive nitrogen species (peroxynitrite) is probably not involved in the antiproliferative effects that Mito_10_-NAC (or its methylated analogs) induced in cancer cells.

We also tested the effects of other NAC analogs (Mito-PEG_4_-NAC and NAC amide) on cell proliferation. The results show that the IC_50_ values at which Mito-PEG_4_-NAC and NAC amide to inhibit cell proliferation are 36.7 µM and 4300 µM, respectively (Fig. 4).

**Figure 5:**
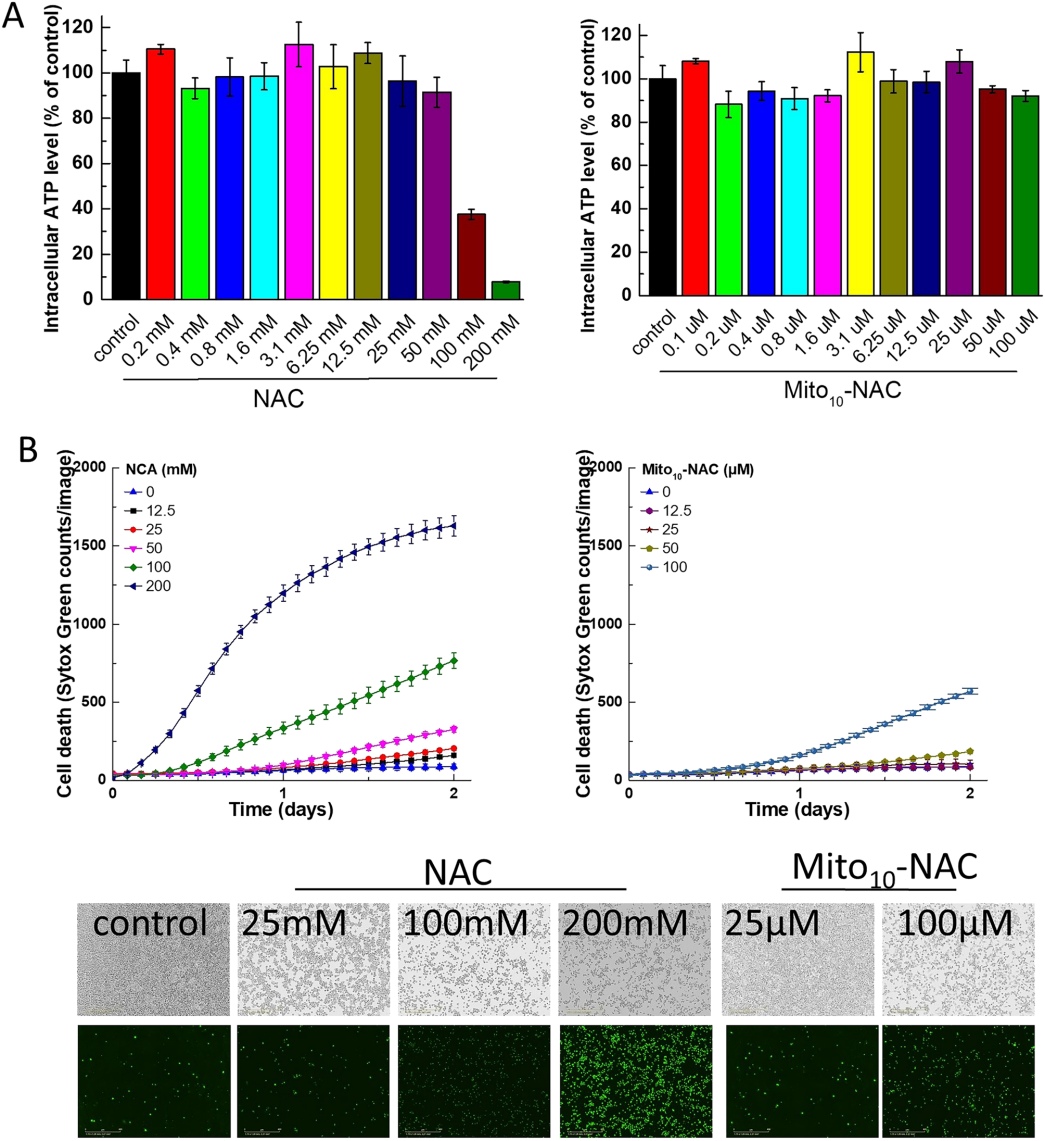
Effects of NAC and Mito_10_-NAC on intracellular ATP levels and cell death in human pancreatic cancer (MiaPaCa-2) cells. (**A**) Effects of NAC and Mito_10_-NAC on the intracellular ATP level. MiaPaCa-2 cells were treated with NAC or Mito_10_-NAC for 24 h, and concentration-dependent inhibition of intracellular ATP level was measured. (**B**) The SYTOX Green assay monitoring the cytotoxicity of NAC and Mito_10_-NAC in MiaPaCa-2 cells. MiaPaCa-2 cells were treated with NAC and Mito_10_-NAC at the indicated concentrations (IC_50_ values from Fig. 6) for 24 h and 48 h. Cell death with strong green fluorescence intensity was monitored with the IncuCyte Live-Cell Analysis System by SYTOX Green staining. The corresponding representative fluorescence images are shown in the left (24 h) and right (48 h) panels. Data shown are the mean ± SD, n = 4.

We monitored both ATP (Fig. 5A) and cell toxicity, as revealed by the SYTOX Green assay (Fig. 5B), in MiaPaCa-2 cells in the presence of NAC and Mito_10_-NAC. At much higher concentrations (> 50 mM), NAC inhibited ATP levels, but there was significant cell death under these conditions. At concentrations inhibiting human pancreatic cancer cell proliferations (i.e., 10–20 μM), Mito_10_-NAC had no effect on ATP or on cell death (Fig. 5A,B).

**Figure 6:**
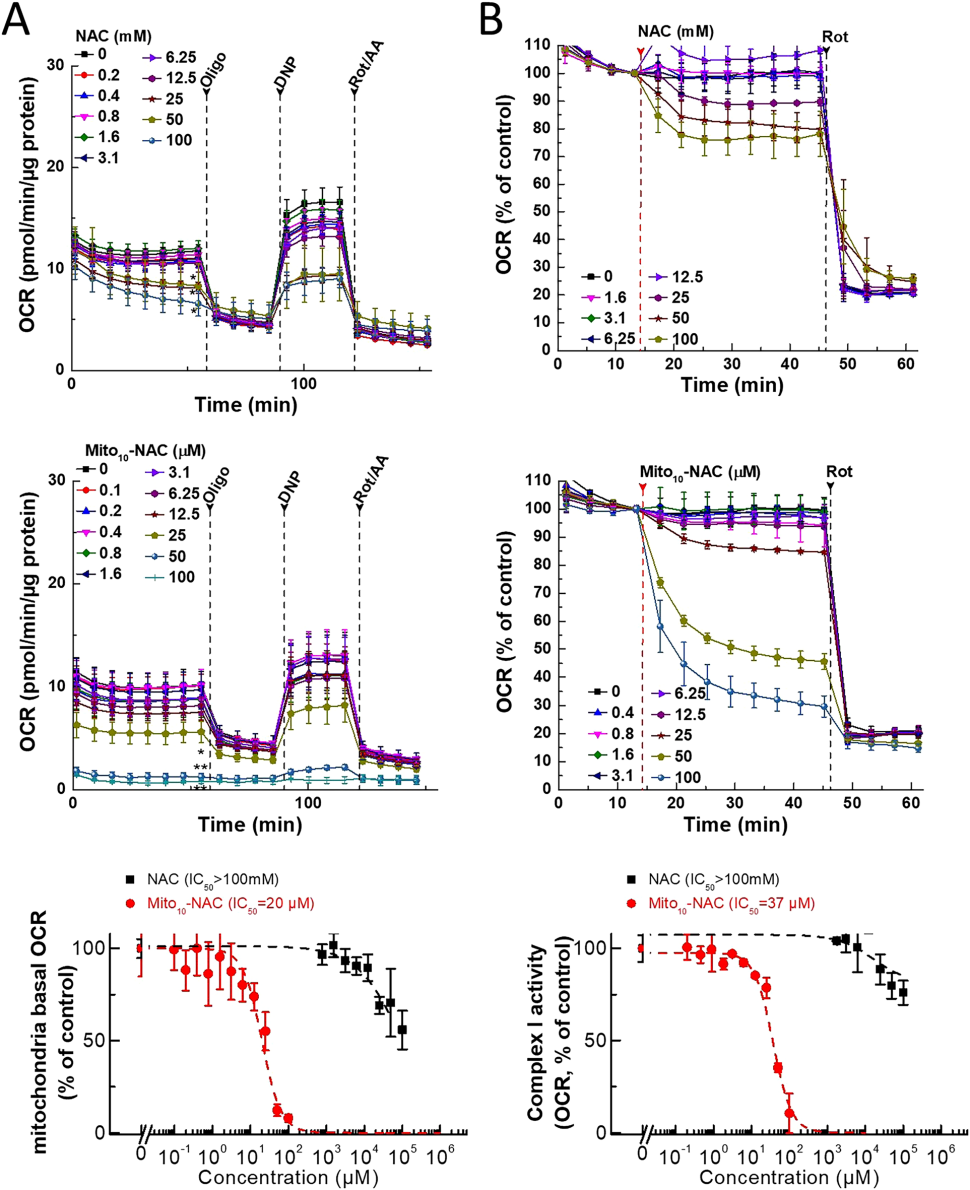
Effects of NAC and Mito_10_-NAC on mitochondria oxygen consumption in either intact cells or by mitochondrial complex I in human pancreatic cancer (MiaPaCa-2) cells. (**A**) Effects of NAC and Mito_10_-NAC on intact cell mitochondria oxygen consumption. MiaPaCa-2 cells were treated with NAC or Mito_10_-NAC for 24 h, and concentration-dependent inhibition of mitochondrial respiration (OCR) in intact MiaPaCa-2 cells was measured. After eight baseline OCR measurements, the response to mitochondrial modulators (oligo, DNP, and rotenone/antimycin A, as described in the “Methods” section) were recorded. *p < 0.05, **p < 0.01 versus control at the last baseline measurements. Data shown are the mean ± SD, n = 4. (**B**) Effects of NAC and Mito_10_-NAC on oxygen consumption by mitochondrial complex I. Dose-dependent effects of NAC or Mito_10_-NAC on complex I-dependent oxygen consumption were measured in permeabilized MiaPaCa-2 cells by direct injection with NAC or Mito_10_-NAC as indicated. Mitochondrial complex I activities were monitored by a Seahorse XF-96 Extracellular Flux Analyzer. Then, Rotenone (complex I inhibitor) was acutely added. The mitochondrial complex I-dependent oxygen consumption was shown and calculated as rotenone inhibitable OCR. The mitochondria basal OCR (A*, bottom*), or mitochondrial complex I dependent OCR direct treatment (B, *bottom*) were plotted against the concentrations of treatments. Dashed lines represent the fitting curves used to determine the IC_50_ values as indicated. Data shown are the mean ± SD, n = 4.

Melanoma (UACC-62) cells behaved quite differently from other cancer cells. Melanoma cells were more resistant to NAC and Mito_10_-NAC, although Mito_10_-NAC was still much more potent than NAC. Another confounding aspect was that NAC exerted a dose-dependent biphasic effect (Fig. S1). At present, we do not have an explanation for the biphasic effect of NAC in the melanoma cells. Due to the experimental limitations of the assays, it was not practical to choose the same time point for cell proliferation or the Seahorse assay and ATP measurements (Figs. 4, 5, and 6).

### The effect of NAC and Mito_10_-NAC on mitochondrial complex I-induced oxygen consumption

Mitochondrial respiration (oxidative phosphorylation [OXPHOS]) and complex I-induced oxygen consumption were assessed using the Seahorse technique^18,19^. MiaPaCa-2 cells were treated with varying concentrations of NAC and Mito_10_-NAC, and the overall oxygen consumption rate (OCR) was measured (Fig. 6A). The usual bioenergetic indices for mitochondrial stress were monitored^19^. As shown, Mito_10_-NAC inhibited 50% of the basal OCR at the 20 µM level, whereas NAC required IC_50_ values at much higher concentrations (> 100 mM) to inhibit the basal OCR.

Because treatment with NAC over a 24 h time period caused considerable cell death (Fig. 5B), we tested to see if NAC or Mito_10_-NAC could directly inhibit mitochondrial complex I-dependent OCR (Fig. 6B) by injecting NAC or Mito_10_-NAC into permeabilized cells in real time. Under these conditions, NAC inhibited only about 30% of complex I-induced oxygen consumption, even up to concentrations of 100 mM; direct cell toxicity is observed at such concentrations (Fig. 5B). In contrast, the IC_50_ value at which Mito_10_-NAC directly inhibits complex I-induced oxygen consumption is 37 µM. At this concentration of Mito_10_-NAC, there was negligible cell toxicity (Fig. 5B). Thiol-based antioxidants (NAC and glutathione [GSH] esters) induced transient mitochondrial oxidation and inhibition of the mitochondrial respiratory complex III in several cancer cells including glioblastoma^25,26^. However, the present results using real-time monitoring of mitochondrial complex I-induced oxygen consumption indicate that NAC had no effect on mitochondrial respiration, even at high concentrations, in MiaPaCa-2 cells.

### The combined effect of NAC/Mito_10_-NAC and Mito_10_-NAC/MCT-1 inhibitor on pancreatic cancer cell proliferation

**Figure 7:**
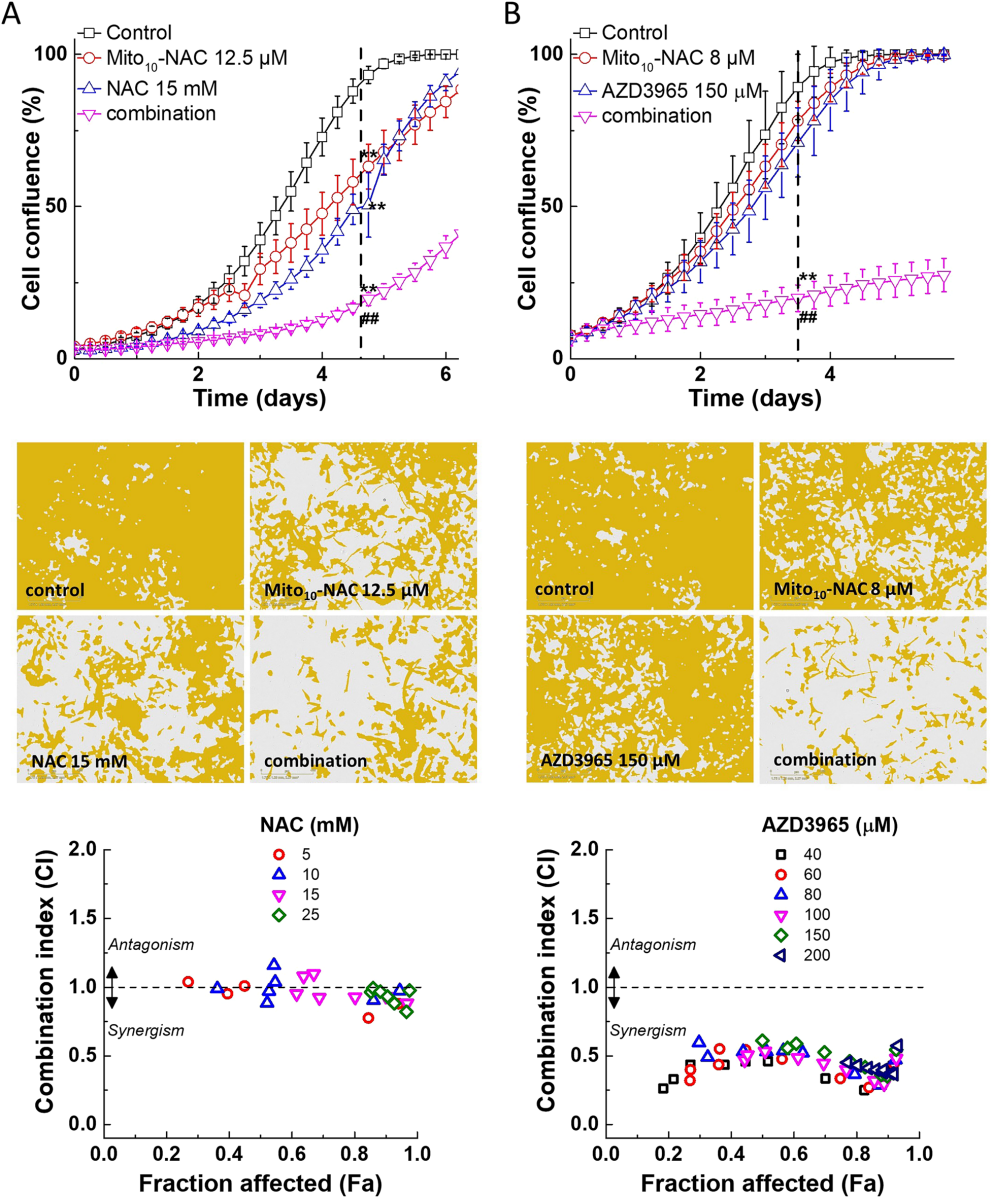
The effects of Mito_10_-NAC in combination with NAC or AZD3965 on inhibition of cell proliferation in human pancreatic cancer (MiaPaCa-2) cells. MiaPaCa-2 cells were treated with Mito_10_-NAC (as indicated) independently or in combination with NAC (**A**, *top and middle*) or AZD3965 (**B**, *top and middle*), and cell growth was monitored continuously. Data shown are the mean ± SD (n = 5). Representative cell images are shown as a segmentation mask illustrated in brown when control cells reached ~ 90% confluence (vertical dashed line). **p < 0.01 vs control. ^##^*p* < 0.01 vs single compound alone. (**A**, **B**, *bottom*) Cell confluence (control cells reached ~ 90% confluence) is plotted against concentration for the synergistic calculation. Panel B shows the combination index-fraction affected plots. Fraction affected parameter is used as a measure of the drug’s efficiency, with a value of 0 indicating complete inhibition of cell confluence and a value of 1 indicating the lack of effect on cell confluence. Mito-NAC concentration range used to calculate confidence interval are 2, 4, 6, 8, 10, 12.5, 15, 25, and 50 µM. Data shown are the mean ± SD, n = 4.

Monocarboxylate transporter has been used as a therapeutic target in cancer cells^27–29^. Previously, we showed that simultaneous inhibition of monocarboxylate transporter 1 (MCT-1) and mitochondrial OXPHOS synergistically inhibited the proliferation of several cancer cells^30^. More recently, these findings were confirmed in a B-cell lymphoma xenograft using AZD3965 and another OXPHOS inhibitor^31^. NAC was reported to inhibit monocarboxylate transporter 4 (MCT-4) expression in cancer cell lines^32^. NAC decreased MCT-4 stromal expression that is used as a biomarker of breast cancer^9,33^. We surmised that Mito_10_-NAC may synergize with NAC. We compared the synergistic effects of Mito_10_-NAC with AZD3965, an MCT-1 inhibitor that is undergoing a Phase I/II clinical trial for cancer therapy. MiaPaCa-2 cells were treated with Mito_10_-NAC and AZD3965 or NAC, independently and together, and cell growth was monitored continuously. These results, presented in Fig. 7, indicate that Mito_10_-NAC is synergistic with MCT-1 inhibitors (AZD3965) but not with NAC, as shown by the combination-index-fraction affected plots.

## Discussion

### Relative inhibitory effects of mitochondria-targeted drugs

We previously reported that increasing the aliphatic chain length in TPP^+^-conjugated molecules greatly enhanced the antiproliferative potencies in tumor cells^18^. As shown in Fig. 3, the fold difference between the parent compound and the TPP^+^-modified compound (with 10 carbons in the linker side chain) is dependent on the parent compound, especially its hydrophobicity. Figure 3 shows the dose response characteristics of NAC, metformin (Met), lonidamine (LND), honokiol (HNK), and atovaquone (ATO) and the TPP^+^-modified analogs in MiaPaCa-2 cells^18,34,35^. The difference in antiproliferative effect between ATO and mitochondria-targeted ATO (Mito-ATO) is 85-fold, whereas the difference between NAC and Mito_10_-NAC is 1600-fold and between Met and Mito_10_-Met is 3300-fold. Although many factors are responsible for the fold difference between the TPP^+^-modified drug and the unmodified drug, the hydrophobicity of the parent drug is a major factor. If the parent compound is very hydrophilic (NAC), TPP^+^ modification will likely induce a greater fold difference in antiproliferative effect and inhibition of mitochondrial respiration in tumor cells^36^ due to the more negative mitochondrial membrane potential of tumor cells as compared with normal cells^20,21,37–39^. We have previously shown that TPP^+^ incorporation to the mitochondria-targeted drug is essential for its mitochondrial accumulation and antiproliferative efficacy in cancer cells^35,36,40^.

### Lack of radical scavenging mechanism

The paradoxical effects of reactive oxygen species (e.g., superoxide and hydrogen peroxide) have previously been reported in cancer cells^12,41–43^. Superoxide and hydrogen peroxide, at low levels, are reported to promote tumorigenesis and tumor progression, but at higher levels, these species induce cytotoxicity in tumor cells and inhibit metastasis^12,43,44^. This suggests that reactive oxygen species inhibition will affect tumorigenesis, tumor progression, and metastasis differently. Redox modulators (NAC) and chain-breaking antioxidant-inhibiting lipid peroxidation (vitamin E) enhanced metastasis of lung cancer in mice^45,46^. However, based on the results obtained with methylated Mito_10_-NAC, we conclude that the reactive oxygen species or redox modulating effects of Mito_10_-NAC are unlikely to play a key role in its antiproliferative mechanism. The IC_50_ value for Mito-NAC-SMe (lacking the –SH group) to inhibit cell proliferation (Fig. 7) is similar or slightly lower than that of Mito-NAC (having the –SH group). Regardless of the presence or absence of the redox-sensitive –SH group, the antiproliferative effects of Mito-NAC and Mito-NAC-SMe are unaffected. We have shown in other studies that blunting the nitroxide moiety (i.e., removing the superoxide dismutase mimetic mechanism) in Mito-CP did not affect its antiproliferative effect^47^. Recently, we showed that reactive oxygen species generation is not responsible for the antiproliferative effects of TPP^+^-based mitochondria-targeted drugs in cancer cells^48^.

### Immunomodulatory effects of NAC and anti-tumor immune function

Recently, NAC has found new applications in immunotherapy^5,6^. Chimeric antigen receptor (CAR) T cells are genetically modified T cells that will recognize and destroy a protein on cancer cells. CAR T cell therapy involves reprogramming a patient’s own T cells to recognize and attack a specific protein in cancer cells, and then infusing the T cells back into the patient^49,50^. Often, enhanced oxidant-induced modifications in CAR T cells decrease this ability. NAC has been shown to improve the efficacy of adoptive T cell immunotherapy to treat melanoma^5,6^. In a recent study, the NAC T cells were cultured before they were infused as immunotherapy in a preclinical model of melanoma; this resulted in an improved outcome^5^. T cells treated with NAC were 33-fold more effective than those cultured without NAC. NAC improves the anti-tumor function of exhausted T cells, thereby enhancing therapeutic outcomes for adoptive cell transfer (ACT) therapy^6^. NAC activates PI3K/Akt, inhibiting Foxo1, and inhibits reactive oxygen species, thereby enhancing the antitumor functionality of T cells^6^. The NAC-mediated opposing effect on T cells was dependent on the concentration. At low concentrations, NAC had an immunostimulatory effect, and at higher concentrations NAC had a suppressive effect^7^. A Phase I clinical trial of NAC, which aims to optimize the metabolic tumor microenvironment, is ongoing^8^. Although the bioavailability of NAC is low, it is membrane-permeable and has been shown to cross the blood–brain barrier in humans and rodents^10,11^.

Because Mito_10_-NAC is considerably more effective in tumor cells, it is of interest to explore the possibility of enhancing CAR T cell therapy using Mito_10_-NAC. Published reports^5,51^ and an ongoing clinical trial^8^ using NAC attest to this possibility. Mitochondria-targeted drugs such as Mito-ATO reprogram the tumor microenvironment, inhibiting tumor-suppressive immune cells and activating T cells^36,48^. Mito_10_-ATO could reverse immunosuppression by regulatory T cells by stimulating the function of effector T cells^48^. Mito-ATO induced potent T cell immune responses in local and distant tumor sites, and it decreased myeloid-derived suppressor cells and regulatory T cells in the tumor microenvironment^52^. Mito-ATO also increased tumor infiltrating CD4^+^ T cells. Mito-ATO improved the efficacy of PD-1 blockade immunotherapy^52^.

### Synergistic antitumor effect of OXPHOS inhibitors and MCT-1/4 inhibitors

The antiproliferative effect of Mito_10_-NAC was enhanced in the presence of AZD3965, an inhibitor of MCT-1 transporter. The extent of the combinatorial effect is consistent with our previously published heat map representation for other mitochondria-targeted drugs^30^. AZD3965 has been reported to enhance intracellular acidosis through increased intracellular lactate and decreased extracellular lactate^53^. Relatively higher concentrations of AZD3965 were used to inhibit cancer cells. At these concentrations, AZD3965 exerts deleterious side effects. The combination therapy with mitochondria-targeted drugs is a promising option as it could significantly lower the concentration of AZD3965 used in cancer therapy. Other mitochondria-targeted drugs (i.e., metformin and phenformin) have been used in combination with AZD3965 in brain cancer studies^54^. However, their effective concentrations were widely different. In this study, Mito_10_-NAC was used at low micromolar levels in combination with AZD3965 to achieve a synergistic inhibition in proliferation.

### Other considerations

The hyperpolarized [1-^13^C] NAC probe of the hyperpolarized ^13^C-MRI showed global distribution of NAC, including to the brain^55^. This finding is consistent with previous studies that used isotopically substituted NAC^56^. Both tumor cell and mice xenograft studies showed that [^13^C] NAC forms a [^13^C] NAC–GSH dimer as well as other homodimers. Further, because no detectable levels of the levels of [^13^C] GSH were found, there is a possibility that NAC could induce GSH synthesis by an indirect mechanism^55^. Additionally, it is suggested that a shortened relaxation time could be the reason no [^13^C] GSH was detected^57^. Induction of GSH by NAC is cell dependent, and the mechanism by which GSH forms has yet to be determined.

The cysteine residue, Cys90, in the ND3 subunit of mitochondrial complex I is reported to play a key role in mitochondrial function^23^. Glutathionylation or nitrosation of this critical cysteine residue regulates redox signaling^58^. Mito_10_-NAC could inhibit complex I by thiolation of mitochondrial cysteine proteome. However, results obtained from using the methylated analog of Mito_10_-NAC indicate that the disruption of mitochondrial cysteine proteome is probably not responsible for Mito_10_-NAC-induced inhibition of mitochondrial respiration. The exact target of Mito_10_-NAC and other analogs in mitochondria still needs to be determined.

It is possible that Mito-NAC-mediated antiproliferative effects are due to cell cycle arrest. Previously, it was reported that mitochondria-targeted drugs (e.g*.*, Mito-magnolol) decreased AKT and Foxo1 phosphorylation and induced cell cycle arrest in the G1 phase of the cell cycle^59^. Future studies investigating the effect of NAC, Mito-NAC, and their methylated analogs on AKT signaling and cell cycle arrest in cancer cells should enhance our understanding of these redox-sensitive thiols.

This study has several limitations. All the experiments were performed in different cancer cell lines. Based on the previous publications in which mitochondrial OXPHOS inhibitors were translated to in vivo mouse xenograft models^18,19,36^, we believe that Mito_10_-NAC and analogs will show similar potency in mouse xenografts as well. Given the current interest in the immunomodulatory effects of NAC, future studies should focus on the effect of Mito_10_-NAC and other related analogs in activated immune cells and immune competent mice. Results indicate that Mito-NAC remained relatively stable over the time course in cell proliferation experiments. However, a comprehensive analytical study is required to monitor any oxidative degradation of compounds over the experimental duration.

## Methods

The methods used in the synthesis, cell experiments, and statistical analysis of Mito_10_-NAC and its analogs follow standard scientific methods we routinely use in our labs, and have been described previously^18,19,22,30,48,60^. Application of these methods to Mito_10_-NAC and its analogs are further described in the subsequent sections.

### Synthesis of Mito_10_-NAC

**Figure 8:**
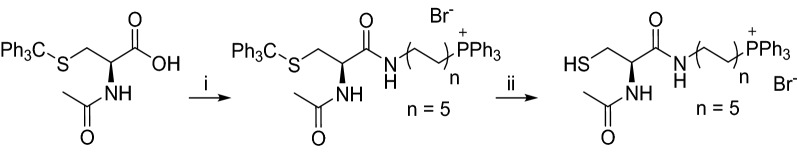
Synthesis of Mito_10_-NAC. Reagents and conditions: i, HOBt, DIC, (10-aminodecyl)-triphenylphosphonium bromide hydrochloride, triethylamine, CH_2_Cl_2_, rt., 12 h, 71%; ii, EtSi-H, TFA, rt., 1 h, 83%.

Mito_10_-NAC was prepared in two steps, by activating the carboxylic acid using *N,N′*-diisopropylcarbodiimide (DIC)/hydroxybenzotriazole (HOBt) followed by the addition of (10-aminodecyl)-triphenylphosphonium bromide in the presence of triethylamine in dichloromethane (CH_2_Cl_2_). Deprotection of thiol by 2,2,2-trifluoroacetic acid (TFA) and triethylsilane delivered the Mito_10_-NAC. The synthesis of Mito_10_-NAC is shown in Fig. 8.

A stirred solution of *N*-acetyl-*S*-trityl-l-cysteine (0.3 g, 0.74 mmoL) in CH_2_Cl_2_/*N*,*N*-dimethylformamide (DMF) (15 mL/100 mL) was cooled to 0 °C and successively treated with HOBt (0.2 g, 1.48 mmoL) and DIC (0.19 g, 1.50 mmoL). After stirring for 2 h at room temperature, (10-aminodecyl)-triphenylphosphonium bromide hydrochloride (0.35 g, 0.65 mmoL) and triethylamine (188 μL, 0.13 mmoL) were added to the mixture. The reaction mixture was stirred overnight at room temperature. Then, CH_2_Cl_2_ was added to the mixture as well as water (H_2_O) (25 mL). The organic layer was dried over sodium sulfate (Na_2_SO_4_). The solvent was removed under reduced pressure. The crude product was poured in 100 mL of ether and centrifuged. The insoluble salt was collected and purified by flash chromatography (CH_2_Cl_2_/ethanol [EtOH] 9/1) and led to the corresponding **trityl-Mito**_**10**_**-NAC** (0.41 g, 71% yield). High-performance liquid chromatography–mass spectrometry (HPLC–MS) indicated that the product was sufficiently pure and could be used without further purification. Electrospray ionization–mass spectrometry (ESI–MS) for **trityl-Mito**_**10**_**-NAC** C_52_H_58_N_2_O_2_PS^+^ [M]^+^, 806.1.

A mixture of **trityl-Mito**_**10**_**-NAC** (0.25 g, 0.31 mmoL), triethylsilane (100 μL, 0.60 mmoL), in trifluoroacetic acid (1 mL) was stirred at room temperature for 1 h. Then, the mixture was purified directly by reverse phase chromatography on a C18 column (H_2_O/acetonitrile [CH_3_CN] from 9/1 to 0/10 with 0.1% of TFA) delivered the corresponding **Mito**_**10**_**-NAC** (0.15 g, 83% yield).

HRMS calculated for **Mito**_**10**_**-NAC** C_33_H_44_N_2_O_2_PS^+^ [M]^+^ 563.2856, found, 563.2856. ^31^P NMR (400.13 MHz, CDCl_3_) δ 23.76. ^1^H NMR (400.13 MHz, CDCl_3_), δ 7.83–7.77 (3H, m), 7.71–7.59 (12H, m), 7.44–7.37 (1H, m), 7.12–7.01 (1H, m), 6.90–6.75 (1H, m), 4.59–4.50 (1H, m), 3.25–3.08 (4H, m), 2.97–2.71 (2H, m), 2.01 (3H, s), 1.58–1.62 (3H, m), 1.52–1.38 (4H, m), 1.26–1.16 (9H, m). ^13^C NMR (75 MHz, CDCl_3_) δ 171.2, 170.2, 135.4, 135.3, 133.4, 133.2, 130.7, 130.5, 118.4, 117.5, 55.2, 39.5, 30.2, 30.1, 28.9, 28.7, 28.6, 28.5, 28.3, 26.7, 26.4, 22.9, 22.4 (d, *J* = 4.4), 22.3 (d, *J* = 51.3).

NMR spectra for Mito_10_-NAC are shown in Fig. S2.

### Synthesis of Mito_10_-NAC-SMe

**Figure 9:**
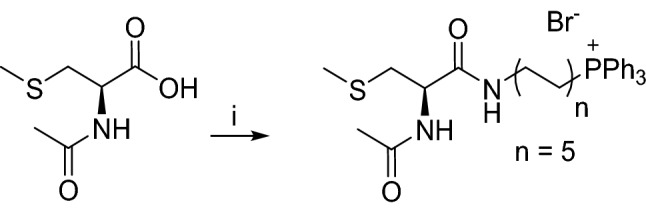
Synthesis of Mito_10_-NAC-SMe. Reagents and conditions: i, HOBt, DIC, (10-aminodecyl)-triphenylphosphonium bromide hydrochloride, triethylamine, CH_2_Cl_2_, rt., 12 h, 43%.

The mitochondria targeted *N*-acetyl methylated cysteine (**Mito**_**10**_**-NAC-SMe**) was prepared using the same reaction conditions as for the synthesis of Mito_10_-NAC. The synthesis of Mito_10_-NAC-SMe is shown in Fig. 9.

A stirred solution of *N*-acetyl-*S*-methyl-l-cysteine (0.15 g, 0.75 mmoL) in CH_2_Cl_2_/DMF (15 mL/100 mL) was cooled to 0 °C and successively treated with HOBt (0.13 g, 0.96 mmoL), DIC (152 µL, 0.96 mmoL). After stirring for 2 h at room temperature, (10-aminodecyl)-triphenylphosphonium bromide hydrochloride (0.35 g, 0.65 mmoL) and triethylamine (188 µL, 0.13 mmoL) were added to the mixture. The reaction mixture was stirred overnight at room temperature. Then, CH_2_Cl_2_ was added to the mixture as well as H_2_O (25 mL). The organic layer was dried over Na_2_SO_4_. The solvent was removed under reduced pressure. The crude product was poured in 100 mL of ether and centrifuged. The insoluble salt was collected and purified by reverse phase chromatography on a C18 column (H_2_O/CH_3_CN from 9/1 to 0/10 with 0.1% of TFA) and delivered the corresponding **Mito**_**10**_**-NAC-SMe** (0.18 g, 43% yield).

HRMS calculated for **Mito**_**10**_**-NAC-SMe** C_34_H_46_N_2_O_2_PS^+^ [M]^+^ 577.3012, found, 577.3015. ^31^P NMR (400.13 MHz, CDCl_3_) δ 23.90. ^1^H NMR (400.13 MHz, CDCl_3_), δ 7.86–7.80 (3H, m), 7.75–7.64 (12H, m), 7.19–7.06 (1H, m), 7.05–6.84 (1H, m), 4.58–4.48 (1H, m), 3.39–3.16 (4H, m), 2.89–2.82 (2H, m), 2.13 (3H, s), 2.04 (3H, s), 1.68–1.42 (6H, m), 1.32–1.19 (10H, m). ^13^C NMR (75 MHz, CDCl_3_) δ 170.8, 170.7, 135.3, 135.2, 133.3, 133.2, 130.6, 130.5, 118.3, 117.5, 52.8, 39.5, 36.4, 30.2, 30.1, 26.5, 22.9, 22.4 (d, *J* = 51.4), 22.3 (d, *J* = 4.4), 15.7.

NMR spectra for Mito_10_-NAC-SMe are shown in Fig. S2.

### Synthesis of Mito-PEG_4_-NAC

**Figure 10:**
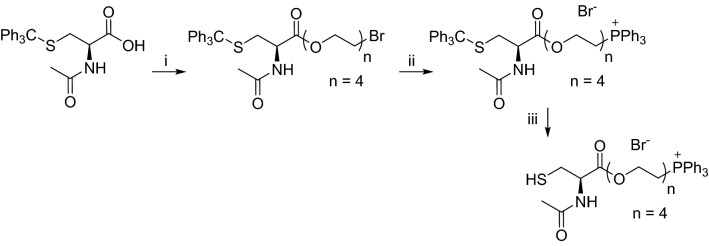
Synthesis of Mito-PEG_4_-NAC. Reagents and conditions: i; HOBt, DIC, (2-[2-[2-(2-bromoethoxy)ethoxy]ethoxy]ethanol, pyridine, CH_2_Cl_2_, rt., 12 h, 58%; ii, PPh_3_, CH_3_CN, reflux, 48 h, 46%; iii, EtSi-H, TFA, rt., 1 h, 95%.

The mitochondria-targeted pegylated *N*-acetylcysteine (Mito-PEG_4_-NAC) was prepared in three steps. Activation of the carboxylic acid by DIC/HOBt followed by the addition of the corresponding bromopegylated derivative in the presence of pyridine in CH_2_Cl_2_ led to the pegylated bromide derivative. The nucleophilic substitution of the bromide by the triphenylphosphine afforded the mitochondria-targeted intermediates. Deprotection of the thiol by TFA and triethylsilane delivered the Mito-PEG_4_-NAC. The synthesis of Mito-PEG_4_-NAC is shown in Fig. 10.

A stirred solution of *N*-acetyl-*S*-trityl-l-cysteine (0.5 g, 1.2 mmoL) in CH_2_Cl_2_ (10 mL) was cooled to 0 °C and successively treated with HOBt (0.3 g, 2.4 mmoL), DIC (390 μL, 2.4 mmoL). After stirring for 2 h at room temperature, (2-[2-[2-(2-bromoethoxy)ethoxy]ethoxy]ethanol (0.28 g, 1.1 mmoL) and pyridine (97 μL, 1.2 mmoL) were added to the mixture. The reaction mixture was then stirred overnight at room temperature. Then, CH_2_Cl_2_ was added to the mixture as well as H_2_O (25 mL). The organic layer was dried over Na_2_SO_4_. The solvent was removed under reduced pressure. The crude product was purified by flash chromatography (CH_2_Cl_2_/EtOH 9/1) and led to the corresponding **trityl-PEG-NAC** (0.46 g, 58% yield). HPLC–MS indicated that the product was sufficiently pure and could be used without further purification. ESI–MS for **trityl-PEG-NAC** C_32_H_38_BrNO_6_S [MH]^+^, 645.0. A mixture of PEG-NAC (0.46 g, 0.7 mmoL) and triphenylphosphine (0.2 g, 0.8 mmoL) in CH3CN was refluxed for 48 h. The crude product was poured into ether. The insoluble salt was purified by flash chromatography (CH_2_Cl_2_/EtOH) and led to the corresponding **trityl-Mito-PEG-NAC** (0.3 g, 46% yield). HPLC–MS indicated that the product was sufficiently pure and can be used without further purification. ESI–MS for **trityl-Mito-PEG-NAC** C_50_H_53_NO_6_PS^+^ [MH]^+^, 826.4.

A mixture of **trityl-Mito-PEG-NAC** (0.2 g, 0.2 mmoL), triethylsilane (a few drops), in trifluoroacetic acid (1 mL) and CH_2_Cl_2_ (1 mL) was stirred at room temperature for 1 h. Then, the mixture was purified directly by reverse phase chromatography on a C18 column (H_2_O/CH_3_CN from 9/1 to 0/10 with 0.1% of TFA) delivered the corresponding **Mito-PEG**_**4**_**-NAC** (0.14 g, 96% yield).

HRMS calculated for **Mito-PEG**_**4**_**-NAC** C_31_H_39_NO_6_PS^+^ [M]^+^ 584.2230, found, 584.2232.

^31^P NMR (400.13 MHz, CDCl_3_) δ 25.09. ^1^H NMR (400.13 MHz, CDCl_3_), δ 7.83–7.63 (15H, m), 6.88–6.77 (1H, m), 6.71–6.62 (1H, m), 4.87–4.78 (1H, m), 4.44–4.19 (2H, m), 3.91–3.65 (6H, m), 3.58–3.52 (2H, m), 3.45–3.40 (2H, m), 3.33 (4H, s), 3.08–2.89 (2H, m), 2.05 (3H, s). ^13^C NMR (75 MHz, CDCl_3_) δ 171.2, 170.1, 134.83, 134.81, 133.9, 133.8, 130.2, 130.0, 119.2, 118.3, 70.5, 70.3, 70.2, 70.0, 68.8, 64.5, 63.6, 63.5, 53.9, 26.7, 24.7 (d, *J* = 53.5), 22.9.

NMR spectra for Mito-PEG_4_-NAC are shown in Fig. S2.

### Cell experiments

#### Cancer cell lines

The following cell lines that were regularly authenticated were obtained from the American Tissue Culture Collection (Manassas, VA): MiaPaCa-2 (Cat# CRL-1420, human pancreatic cancer cells), MDA-MB-231(Cat# HTB-26, human breast cancer cells), and MCF-7 (Cat# HTB-22, human breast cancer cells). The UACC-62 melanoma cell line was purchased from AddexBio (San Diego, CA; Cat# C0020003) where it was regularly authenticated. All cell lines were grown at 37 °C in 5% carbon dioxide. MiaPaCa-2 and MDA-MB-231 cells were maintained in DMEM medium (Thermo Fisher Scientific, Cat# 11965) and supplemented with 10% fetal bovine serum. MCF-7 cells were maintained in MEM-α (Thermo Fisher Scientific, Cat# 12571) containing 10% fetal bovine serum. UACC-62 cells were maintained in RPMI 1640 medium (Thermo Fisher Scientific, Cat# 11875) and supplemented with 10% fetal bovine serum. All cells were stored in liquid nitrogen and used within 20 passages after thawing.

#### Cell proliferation measurements

The IncyCyte Live-Cell Analysis System was used to monitor cell proliferation^22,60^. This imaging system is noninvasive and enables continuous monitoring of cell confluence over several days. In a 96-well plate, cells were plated at 1000 cells per well in triplicates and left to adhere overnight. Cells were then treated with compounds tested at indicated concentrations, and the cell confluency was recorded over several days in the IncuCyte Live-Cell Analysis System.

#### Intracellular ATP levels

After seeding cells, overnight at 20,000 per well in 96-well plates, cells were exposed to NAC analogs for 24 h. A luciferase-based assay was used to measure intracellular ATP levels as per the manufacturer’s instructions (Sigma Aldrich, St. Louis, MO, Cat# FLAA). Briefly, an ATP assay mix solution consisting of luciferase and luciferin (Cat# FLAAM) was added to cell lysates. After swirling, the amount of light produced was immediately recorded in a luminometer. The results were normalized to the total protein level measured in each well, as determined by the Bradford method (Bio-Rad Laboratories, Hercules, CA).

#### Mitochondrial respiration measurements

Mitochondrial oxygen consumption was measured in the Seahorse XF-96 Extracelluar Flux Analyzer (Agilent, North Billerica, MA)^18,19,48^. The bioenergetic function assay was used to determine the intact cell mitochondrial function of cells in response to drug treatment^18,19,48^. After cells were treated with NAC or Mito_10_-NAC for 24 h, eight baseline OCR measurements were taken before injection of oligomycin (1 mg/mL) to inhibit ATP synthase, dinitrophenol (50 µM) to uncouple the mitochondria and yield maximal OCR, and inhibitors of complexes I and III (1 µM rotenone and antimycin) to inhibit mitochondrial respiration. From these measurements, mitochondrial function indices were determined^18,19,48^.

For mitochondrial complex I activity measurements, the mitochondrial complex I-induced OCR measurements were carried out in permeabilized cells in the presence of complex I substrates pyruvate/malate and complex II inhibitor malonate (10 mM)^18,19,48^. The IC_50_ values were determined as previously reported^19,30,48^.

### Statistical analysis

Comparisons between the control and treatment groups were made using an unpaired Student’s t-test analysis. *P* values of less than 0.05 were considered to be statistically significant. All values provided represent mean ± standard deviation. The number of replicates per treatment group are shown as *n*. The IC_50_ values and fitting curves were calculated using OriginPro 2016 (OriginLab Corporation, Northampton, MA).

## Supplementary Information

Supplementary Figures.

## Data Availability

This study did not generate/analyze any computational datasets/code nor publicly archived datasets.

## References

[1] Schwalfenberg GK (2021). N-Acetylcysteine: A review of clinical usefulness (an old drug with new tricks). J. Nutr. Metab..

[2] Schwaiger, T. *A Review of the Use of N-Acetyl-Cysteine (NAC) in Clinical Practice*, https://www.naturalmedicinejournal.com/journal/review-use-n-acetyl-cysteine-nac-clinical-practice (2021).

[3] Kwon Y (2021). Possible beneficial effects of N-acetylcysteine for treatment of triple-negative breast cancer. Antioxidants.

[4] Feng H (2021). N-acetyl cysteine induces quiescent-like pancreatic stellate cells from an active state and attenuates cancer-stroma interactions. J. Exp. Clin. Cancer Res..

[5] Scheffel MJ (2016). Efficacy of adoptive T-cell therapy is improved by treatment with the antioxidant N-acetyl cysteine, which limits activation-induced T-cell death. Cancer Res..

[6] Scheffel MJ (2018). N-acetyl cysteine protects anti-melanoma cytotoxic T cells from exhaustion induced by rapid expansion via the downmodulation of Foxo1 in an Akt-dependent manner. Cancer Immunol. Immunother..

[7] Karlsson H (2009). *N-acetyl-L*-Cysteine Promotes T Cell Mediated Immunity In Allogeneic Settings *IN VIVO* And *IN VITRO*. Am. Soc. Blood Marrow Transpl..

[8] Memorial Sloan Kettering Cancer Center. *A Study of N-Acetylcysteine (N-AC)in People Receiving CAR T-cell Therapy for Lymphoma*, https://www.clinicaltrials.gov/ct2/show/NCT05081479 (2022).

[9] Monti D (2017). Pilot study demonstrating metabolic and anti-proliferative effects of in vivo anti-oxidant supplementation with N-Acetylcysteine in Breast Cancer. Semin. Oncol..

[10] Olsson B, Johansson M, Gabrielsson J, Bolme P (1988). Pharmacokinetics and bioavailability of reduced and oxidized N-acetylcysteine. Eur. J. Clin. Pharmacol..

[11] Holdiness MR (1991). Clinical pharmacokinetics of N-acetylcysteine. Clin. Pharmacokinet.

[12] Chio IIC, Tuveson DA (2017). ROS in cancer: The burning question. Trends Mol. Med..

[13] Hara Y, McKeehan N, Dacks PA, Fillit HM (2017). Evaluation of the neuroprotective potential of N-acetylcysteine for prevention and treatment of cognitive aging and dementia. J. Prev. Alzheimers Dis..

[14] Bavarsad Shahripour R, Harrigan MR, Alexandrov AV (2014). N-acetylcysteine (NAC) in neurological disorders: Mechanisms of action and therapeutic opportunities. Brain Behav..

[15] Aldini G (2018). N-Acetylcysteine as an antioxidant and disulphide breaking agent: The reasons why. Free Radic. Res..

[16] Bansal A, Simon MC (2018). Glutathione metabolism in cancer progression and treatment resistance. J. Cell Biol..

[17] Yim CY, Hibbs JB, McGregor JR, Galinsky RE, Samlowski WE (1994). Use of N-acetyl cysteine to increase intracellular glutathione during the induction of antitumor responses by IL-2. J. Immunol..

[18] Cheng G (2016). Mitochondria-targeted analogues of metformin exhibit enhanced antiproliferative and radiosensitizing effects in pancreatic cancer cells. Cancer Res..

[19] Cheng G (2019). Targeting lonidamine to mitochondria mitigates lung tumorigenesis and brain metastasis. Nat. Commun..

[20] Zielonka J (2017). Mitochondria-targeted triphenylphosphonium-based compounds: Syntheses, mechanisms of action, and therapeutic and diagnostic applications. Chem. Rev..

[21] Murphy MP, Hartley RC (2018). Mitochondria as a therapeutic target for common pathologies. Nat. Rev. Drug Discov..

[22] Cheng G (2013). Mitochondria-targeted vitamin *E. analogs* inhibit breast cancer cell energy metabolism and promote cell death. BMC Cancer.

[23] Bak DW, Pizzagalli MD, Weerapana E (2017). Identifying functional cysteine residues in the mitochondria. ACS Chem. Biol..

[24] Hughes CE (2020). Cysteine toxicity drives age-related mitochondrial decline by altering iron homeostasis. Cell.

[25] Beaudoin JN (2013). Thiol-based antioxidants trigger transient mitochondrial oxidation. FASEB J..

[26] Kolossov VL (2015). Thiol-based antioxidants elicit mitochondrial oxidation via respiratory complex III. Am. J. Physiol. Cell Physiol..

[27] Baltazar F (2014). Monocarboxylate transporters as targets and mediators in cancer therapy response. Histol. Histopathol..

[28] Jones RS, Morris ME (2016). Monocarboxylate transporters: Therapeutic targets and prognostic factors in disease. Clin. Pharmacol. Ther..

[29] Payen VL, Mina E, Van Hée VF, Porporato PE, Sonveaux P (2020). Monocarboxylate transporters in cancer. Mol. Metab..

[30] Cheng G, Hardy M, You M, Kalyanaraman B (2022). Combining PEGylated mito-atovaquone with MCT and Krebs cycle redox inhibitors as a potential strategy to abrogate tumor cell proliferation. Sci. Rep..

[31] Noble RA (2022). Simultaneous targeting of glycolysis and oxidative phosphorylation as a therapeutic strategy to treat diffuse large B-cell lymphoma. Br. J. Cancer.

[32] Takenaga K (2021). MCT4 is induced by metastasis-enhancing pathogenic mitochondrial NADH dehydrogenase gene mutations and can be a therapeutic target. Sci. Rep..

[33] Martinez-Outschoorn UE (2013). Oncogenes and inflammation rewire host energy metabolism in the tumor microenvironment: RAS and NFκB target stromal MCT4. Cell Cycle.

[34] Boulware DR (2020). A randomized trial of hydroxychloroquine as postexposure prophylaxis for covid-19. N. Engl. J. Med..

[35] Pan J (2018). Mitochondria-targeted Honokiol Confers a striking inhibitory effect on lung cancer via inhibiting complex I activity. iScience.

[36] Cheng G (2021). Mitochondria-targeted hydroxyurea inhibits OXPHOS and induces antiproliferative and immunomodulatory effects. iScience.

[37] Crunkhorn S (2021). Targeting the mitochondria to block tumour growth. Nat. Rev. Drug Discov..

[38] Zorova LD (2018). Mitochondrial membrane potential. Anal. Biochem..

[39] Dong L, Neuzil J (2019). Targeting mitochondria as an anticancer strategy. Cancer Commun..

[40] Cheng G (2012). Mitochondria-targeted drugs synergize with 2-deoxyglucose to trigger breast cancer cell death. Cancer Res..

[41] Nakamura H, Takada K (2021). Reactive oxygen species in cancer: Current findings and future directions. Cancer Sci..

[42] Sullivan LB, Chandel NS (2014). Mitochondrial reactive oxygen species and cancer. Cancer Metab..

[43] Gill JG, Piskounova E, Morrison SJ (2016). Cancer, oxidative stress, and metastasis. Cold Spring Harb. Symp. Quant. Biol..

[44] Kong H, Chandel NS (2018). Regulation of redox balance in cancer and T cells. J. Biol. Chem..

[45] Sayin VI (2014). Antioxidants accelerate lung cancer progression in mice. Sci. Transl. Med..

[46] Breau M (2019). The antioxidant N-acetylcysteine protects from lung emphysema but induces lung adenocarcinoma in mice. JCI Insight.

[47] Cheng G (2015). Antiproliferative effects of mitochondria-targeted cationic antioxidants and analogs: Role of mitochondrial bioenergetics and energy-sensing mechanism. Cancer Lett..

[48] Cheng G (2020). Potent inhibition of tumour cell proliferation and immunoregulatory function by mitochondria-targeted atovaquone. Sci. Rep..

[49] June CH, O'Connor RS, Kawalekar OU, Ghassemi S, Milone MC (2018). CAR T cell immunotherapy for human cancer. Science.

[50] Sterner RC, Sterner RM (2021). CAR-T cell therapy: Current limitations and potential strategies. Blood Cancer J..

[51] Kalyanaraman B (2022). NAC, NAC, Knockin' on Heaven's door: Interpreting the mechanism of action of N-acetylcysteine in tumor and immune cells. Redox Biol..

[52] Huang M (2022). Prevention of tumor growth and dissemination by in situ vaccination with mitochondria-targeted atovaquone. Adv. Sci..

[53] Pérez-Escuredo J (2016). Monocarboxylate transporters in the brain and in cancer. Biochim. Biophys. Acta.

[54] Beloueche-Babari M (2017). MCT1 Inhibitor AZD3965 increases mitochondrial metabolism, facilitating combination therapy and noninvasive magnetic resonance spectroscopy. Cancer Res..

[55] Yamamoto K (2021). Real-Time insight into in vivo redox status utilizing hyperpolarized [1-(13)C] N-acetyl cysteine. Sci. Rep..

[56] Lauterburg BH, Corcoran GB, Mitchell JR (1983). Mechanism of action of N-acetylcysteine in the protection against the hepatotoxicity of acetaminophen in rats in vivo. J. Clin. Invest..

[57] Onukwufor JO (2022). A reversible mitochondrial complex I thiol switch mediates hypoxic avoidance behavior in *C. elegans*. Nat. Commun..

[58] Go YM, Chandler JD, Jones DP (2015). The cysteine proteome. Free Radic. Biol. Med..

[59] AbuEid M (2021). Synchronous effects of targeted mitochondrial complex I inhibitors on tumor and immune cells abrogate melanoma progression. iScience.

[60] Boyle KA (2018). Mitochondria-targeted drugs stimulate mitophagy and abrogate colon cancer cell proliferation. J. Biol. Chem..

