# Supplementary material for: Antiproliferative effects of mitochondria-targeted N-acetylcysteine and analogs in cancer cells
Source: Sci Rep. 2023 May 4;13:7254. doi: 10.1038/s41598-023-34266-w (PMC10160116; doi:10.1038/s41598-023-34266-w)
Supplement: Supplementary file 1 — Supplementary Figures. [file 41598_2023_34266_MOESM1_ESM.docx]

**Antiproliferative Effects of Mitochondria-Targeted N-acetylcysteine and Analogs in Cancer Cells**

Gang Cheng^1^, Micael Hardy^2^, Balaraman Kalyanaraman^1^

^1^Department of Biophysics, Medical College of Wisconsin, 8701 Watertown Plank Road, Milwaukee, WI 53226, United States

^3^Aix Marseille Univ, CNRS, ICR, UMR 7273, Marseille 13013, France

^*^Correspondence: balarama@mcw.edu

**Supplementary Information**


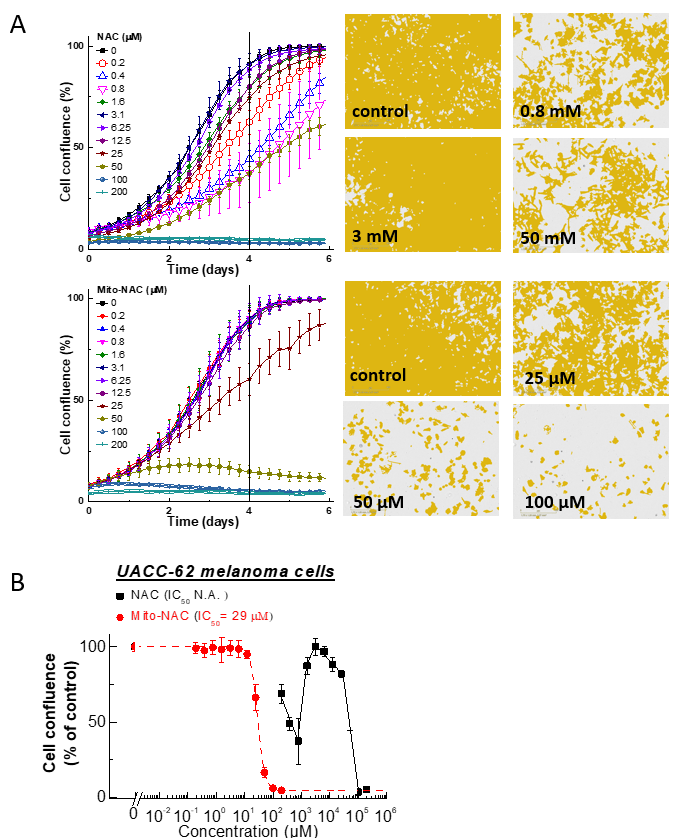


**Figure S1. Effects of NAC and Mito_10_-NAC on the proliferation of melanoma cancer (UACC-62) cells. (A)** The effects of NAC and Mito_10_-NAC on the proliferation of UACC-62 cells were monitored in the IncuCyte Live-Cell Analysis system. The IncuCyte analyzer provides real-time updates on cell confluence, based on segmentation of high definition-phase contrast images. Representative cell images were shown as segmentation mask illustrated in brown when control cells reached 90% confluence (vertical solid black line). **(B)** The IC_50_ values were determined at the point at which control cells reached ~90% confluence. Relative cell confluence (control is taken as 100%) is plotted against concentration. Dashed lines represent the fitting curves used to determine the IC_50_ values as indicated. Data shown are the mean±SD, n=4.

NMR Spectra of **Mito_10_-NAC**.

^13^P NMR (400.13 MHz, CDCl_3_)

^1^H NMR (400.13 MHz, CDCl_3_)

^13^CAPT (75 MHz, CDCl_3_)

NMR Spectra of **Mito_10_-NAC-SMe**.

^13^P NMR (400.13 MHz, CDCl_3_)

^1^H NMR (400.13 MHz, CDCl_3_)

^13^CAPT (75 MHz, CDCl_3_)

NMR Spectra of **Mito-PEG_4_-NAC**.

^13^P NMR (400.13 MHz, CDCl_3_)

^1^H NMR (400.13 MHz, CDCl_3_)

^13^CAPT (75 MHz, CDCl_3_)

**Figure S2.** **NMR Spectra.**

**
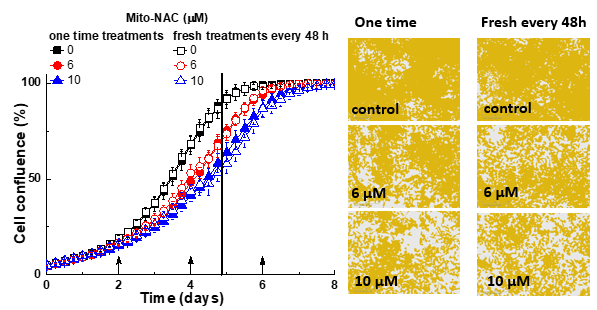
**

**Figure S3. Effects of Mito-NAC on the proliferation of pancreatic cancer (MiaPaCa-2) cells with different treatment schedules.** The effects of Mito-NAC on the proliferation of MiaPaCa-2 cells were monitored in the IncuCyte Live-Cell Analysis system. MiaPaCa-2 cells were treated with Mito-NAC either only once at the beginning of the experiment or received a fresh treatment every 48 h as indicated. Representative cell images were shown as segmentation mask illustrated in brown when control cells reached 90% confluence (vertical solid black line). No significant different between two scheduled treatment at the same concentration. Data shown are the mean±SD, n=4.
